# Study on Molecular Beam Epitaxy Grown HgCdTe Heterojunction Materials

**DOI:** 10.3390/ma19194091

**Published:** 2026-09-24

**Authors:** Weirong Xing, Dan Wang, Mengjia Jiang, Ming Liu, Qing Wu, Songlin Yu, Yongqing Peng, Ruzhi Wang

**Affiliations:** 1 State Key Laboratory of Materials Low-Carbon Recycling, College of Materials Science and Engineering, Beijing University of Technology, Beijing 100124, China; 2 North China Research Institute of Electro-Optics, Beijing 100015, China; wd2320900729@126.com (D.W.); 13920585519@163.com (M.J.); 2023040044@bupt.cn (M.L.); wuqingcg@163.com (Q.W.); yusir8511@sina.com (S.Y.); 3 Beijing Institute of Telemetry Technology, Beijing 100076, China; pengyongqing1980@163.com

**Keywords:** HgCdTe, As doping, annealing activation, heterojunction, high operating temperature

## Abstract

With the development of application requirements for optoelectronic systems, infrared detectors are required to evolve toward smaller size, lighter weight, and lower power consumption, known as “SWaP^3^”. Mercury cadmium telluride (HgCdTe) has become an important research subject for high-temperature infrared detectors due to its inherent material properties. HgCdTe heterojunction materials can reduce detector dark current and elevate the operating temperature of detectors. In situ arsenic-doped HgCdTe grown by molecular beam epitaxy (MBE) serves as the foundation for fabricating low-dark-current, high-operating-temperature detectors. This work investigates the effects of arsenic beam flux and growth temperature on arsenic doping in HgCdTe. A two-step annealing method was employed to study arsenic activation, revealing that doping concentration and annealing atmosphere significantly influence the activation rate and carrier mobility. When the doping concentration is below 5 × 10^17^ cm^−3^, the arsenic activation rate approaches 100%. Annealing under a mixed atmosphere reduces material defects in HgCdTe and markedly improves the arsenic activation rate and carrier mobility. Si-based HgCdTe heterojunction materials were prepared using MBE, and mid-wave HgCdTe infrared detectors were fabricated based on these epitaxial materials. The test results show that the detector operating temperature is greatly increased from 77 K to 110 K, which can significantly reduce detector cost and improve reliability. The results provide significant guidance for the preparation of MBE-grown, arsenic-doped HgCdTe heterojunction materials and lay a solid foundation for the engineering of low-cost, high-operating-temperature HgCdTe infrared detectors.

## 1. Introduction

Mercury cadmium telluride (HgCdTe) features a tunable bandgap; it is capable of detecting infrared radiation ranging from 1 μm to 20 μm and exhibits high quantum efficiency and excellent photoelectric response characteristics, making it a widely used infrared material [1,2], such as for detectors in Fourier-transform infrared (FTIR) spectroscopy and infrared thermal imaging. As the demand for the high-temperature operation of infrared focal plane arrays in optoelectronic systems becomes increasingly urgent, HgCdTe detectors require optimized material structures to improve performance at elevated operating temperatures.

Conventional boron-implanted n-on-p heterojunctions utilize mercury vacancies in HgCdTe to form an intrinsic p-type layer; however, mercury vacancies tend to introduce recombination centers and reduce carrier lifetime. In contrast, in situ doped p-on-n heterojunctions offer superior advantages: low-concentration n-type doping can effectively reduce the generation–recombination current, increase minority carrier lifetime, and overcome the inherent drawbacks of conventional mercury vacancy doping. HgCdTe heterojunction materials can reduce detector dark current and elevate the detector operating temperature [3,4]. Doping of HgCdTe is a prerequisite for realizing heterojunction structures. To meet the design requirements of various detector structures, carrier concentration needs to be controlled over a wide range. Molecular beam epitaxy (MBE) in situ doping technology enables both n-type and p-type doping of HgCdTe and supports multilayer structure design [5,6,7]. In situ doping technology for HgCdTe is a key technology for achieving high-performance infrared focal plane arrays and remains a focus of current research.

Arsenic (As) exhibits low diffusivity and good thermal stability in HgCdTe, making it the preferred element for p-type doping [1,8]. Since it is experimentally difficult to directly observe the migration path of As atoms from Hg sites to Te sites, or to characterize the microstructure of intermediate defects during migration, researchers generally rely on the theoretical framework established by Berding [9] to analyze the p-type activation mechanism of As doping in HgCdTe. In recent years, numerous investigators have conducted in-depth studies on arsenic doping, advancing understanding of this field. Klein et al. [10] elucidated the regulation of As_2_/As_4_ beam flux and the Te-site preferential doping mechanism; Baker et al. [11] accurately determined the As acceptor level at 19.5 meV, refining the narrow-bandgap doping theory model; and Garcia et al. [12] revealed the Hg-vacancy-assisted As diffusion mechanism and established a partial-pressure-controlled diffusion model, in which As initially occupies Hg sites to form As_Hg_ defects, then replaces Te atoms near vacancies, while Te atoms occupy mercury vacancies to form Te_Hg_ antisite defects [9,13]. In 2022, the Harris group [14] proposed superlattice confinement to suppress As diffusion and fabricated atomically abrupt PN junctions, providing a basis for the subsequent development of very-long-wavelength focal plane devices. Although the principles of arsenic doping have been studied above, a systematic description of the preparation of arsenic-doped HgCdTe materials is still lacking.

Accordingly, this paper investigates the effects of arsenic beam flux and HgCdTe growth temperature on doping concentration during epitaxial growth, and studies the effects of arsenic doping concentration and different annealing atmospheres on arsenic activation. Furthermore, HgCdTe detectors were fabricated using heterojunction epitaxial materials, providing a reference for the engineering preparation of low-cost, high-reliability HgCdTe detectors. Silicon substrates are preferred as epitaxial substrates due to their higher mechanical strength, lower cost, capability for larger-size material growth, and good compatibility with detector readout circuits.

## 2. Experiment

Substrate cleaning: 3-inch Si (211) substrates were cleaned in two steps. Trichloroethylene (CAS: 79-01-6), acetone (CAS: 67-64-1, 99%), and anhydrous ethanol (CAS: 64-17-5, 99.5%) were used to remove surface organic contaminants. Ammonia water (CAS: 1336-21-6, 28–30 wt%), aqueous hydrogen peroxide (CAS: 7722-84-1, 31 ± 1 wt%), hydrochloric acid (CAS: 7647-01-0, 36–38 wt%), and hydrofluoric acid (CAS: 7664-39-3, 40–42 wt%) were adopted to remove surface inorganic contaminants.

Substrate pretreatment: The cleaned Si substrates were loaded into the MBE system (DCA, Turku, Finland). In the load–lock chamber, the substrates were heated at 180 °C for 1 h to remove moisture; when the chamber pressure dropped below 5 × 10^−7^ Torr, the samples were transferred to the pretreatment chamber. The samples were heated at 400 °C for 1 h in the pretreatment chamber to remove surface organic matter. Then, the substrates were transferred to the growth chamber once the pretreatment chamber pressure was lower than 2 × 10^−8^ Torr. When the substrates were transferred into the growth chamber, the pressure was lower than 1 × 10^−10^ Torr; then the substrates were heated to approximately 700 °C in the chamber to remove surface oxides, facilitating subsequent high-crystal-quality thin film growth.

Buffer layer growth: After removing the oxide layer, the substrate temperature was lowered to approximately 400 °C. A beam flux monitor (BFM) was used to measure and calibrate the beam fluxes of CdTe, Te, and other effusion sources to achieve appropriate ratios, with a measurement error of 1 × 10^−9^ Torr. The source shutters were opened to grow a 6 μm CdTe buffer layer, which serves to balance the strain caused by the large lattice mismatch between the Si substrate and HgCdTe, reduce the dislocation density in subsequently grown HgCdTe [15,16], and improve the crystalline quality of the material.

Growth of in situ doped HgCdTe: The substrate with the CdTe buffer layer was transferred to another growth chamber for HgCdTe epitaxial layer growth, with simultaneous in situ As doping, as shown in Figure 1. The BFM was used to calibrate the beam fluxes of CdTe, Te, and the Hg source to achieve appropriate ratios. The source shutters were opened to grow HgCdTe, while the doping source shutter was opened simultaneously to incorporate arsenic into HgCdTe, achieving in situ doping during epitaxy, as shown in Figure 2.

Annealing activation sample preparation: The in situ As-doped HgCdTe materials were removed from the growth system and diced into 20 mm × 25 mm pieces. The pieces were sealed in quartz ampoules and then annealed in a furnace at preset temperatures and durations, followed by natural cooling after completion.

Scanning electron microscopy (SEM, Thermo Fisher Scientific, Waltham, MA, USA) was used to observe the surface morphology of the epitaxial materials; the characterization was performed at an accelerating voltage of 5 kV with a beam current of 1.6 nA. Secondary ion mass spectrometry (SIMS) was employed to measure the As doping concentration. Hall measurements were performed to characterize the carrier concentration, mobility, and conductivity type of As-doped HgCdTe before and after activation. Hall-effect measurements were carried out using Van der Pauw geometry; the measurement error of carrier concentration was 1.99%, and mobility was 2.36%.

In addition, we uniformly define the key parameters throughout the manuscript: (1) the doping concentration: the total arsenic atomic concentration measured using SIMS; (2) the carrier concentration: the net electrically active carrier concentration obtained from Hall measurement; and (3) the activation rate: the ratio of the active carrier concentration to the total arsenic doping concentration.

## 3. Results and Discussion

### 3.1. Regulation of As Doping Concentration

During MBE growth, arsenic doping is achieved by sputtering arsenic from a cracker source onto the HgCdTe surface. The cracker temperature affects the molecular form of the emitted arsenic. Studies have shown that the arsenic incorporation mode is highly dependent on the mercury pressure and arsenic beam flux used during growth; arsenic may be incorporated as isolated atoms or as neutral/donor-complex tetramers and clusters [17,18]. During MBE in situ doping, the incorporation efficiency of As_2_ is substantially higher than that of As_4_ [19]. Therefore, in this work, the cracking zone temperature of the arsenic cracker source was set to 900 °C during epitaxial growth to ensure that arsenic was incorporated into HgCdTe in the form of As_2_. The arsenic beam flux was controlled by adjusting the needle valve position of the arsenic source, thereby regulating the amount of arsenic incorporated into HgCdTe. The arsenic beam flux at different needle valve positions was measured, and the corresponding relationship was obtained. As shown in Figure 3, when the needle valve opening is 10%, the arsenic beam flux is 3.5 × 10^−8^ Torr; at 50% opening, it is approximately 5.5 × 10^−8^ Torr; and at 90% opening, it reaches 7.5 × 10^−8^ Torr. Overall, the arsenic source needle valve position exhibits a roughly linear relationship with the arsenic beam flux, which provides guidance for subsequent regulation of arsenic doping concentration.

Previous studies have shown that the arsenic beam flux from the MBE arsenic cracker source exhibits the best stability when the Tank zone temperature is 250 °C. Accordingly, the arsenic source was operated with the Tank zone at 250 °C and the cracking zone at 900 °C. An HgCdTe layer with a total thickness of 8 μm was grown by varying the needle valve position. The initial 1.5 μm was grown without opening the arsenic needle valve, followed by growth at needle valve openings of 10%, 20%, 40%, and 50% (openings exceeding 50% caused beam flux instability). Secondary ion mass spectrometry (SIMS) measurements yielded corresponding arsenic doping concentrations of 2 × 10^17^ cm^−3^, 4 × 10^17^ cm^−3^, 1 × 10^18^ cm^−3^, and 3 × 10^18^ cm^−3^, respectively. The results are shown in Figure 4. With the HgCdTe composition and growth temperature kept constant, the As doping concentration increases with the increasing As beam flux; however, the relationship between doping concentration and the needle valve position/beam flux is not linear.

Varying the arsenic beam flux is the primary means of regulating doping in HgCdTe. However, since the sticking coefficient of arsenic on the substrate surface is temperature-dependent [20], precise control of the incorporation concentration requires further investigation of the effect of the growth temperature on the doping concentration. In another experiment, the As doping source conditions (Tank zone 250 °C and cracking zone 900 °C) and the HgCdTe composition were fixed, and three HgCdTe epitaxial wafers were grown at different temperatures (Table 1) to investigate the effect of the growth temperature on the doping concentration.

Figure 5 shows SIMS profiles of As-doped HgCdTe grown at different temperatures. For growth temperatures of 168 °C, 163 °C, and 161 °C, the doping concentrations are 3 × 10^16^ cm^−3^, 5 × 10^17^ cm^−3^, and 2 × 10^19^ cm^−3^, respectively. Higher growth temperatures result in lower As doping concentrations. With increasing substrate temperatures, the surface sticking coefficient of arsenic decreases remarkably, leading to a sharp reduction in arsenic incorporation within HgCdTe epilayers during MBE growth [21,22]. This further demonstrates the influence of material temperature on the doping concentration during growth. Therefore, during the growth of As-doped HgCdTe, the temperature consistency of the epitaxial material over time must be maintained to ensure uniform longitudinal doping. For large-size material growth, temperature uniformity between the edge and the center of the wafer must also be maintained to ensure lateral doping concentration uniformity and avoid degradation of the device performance.

### 3.2. As Doping Activation

To thoroughly analyze the electrical properties of arsenic atoms in HgCdTe, Hall measurements were performed on as-grown samples without annealing at 77 K. The Hall measurement results are presented in Table 2. Sample 4# is the intrinsic growth control group; Hall testing shows p-type conductivity with a carrier concentration of 4.86 × 10^14^ cm^−3^ and a mobility of 356 cm^2^/Vs. Since MBE-grown HgCdTe is grown under Te-rich conditions, the as-grown wafer exhibits p-type conductivity dominated by mercury vacancies. In contrast, the As-doped samples 5#, 6#, and 7# show n-type conductivity in their Hall measurements, which has been attributed to defects other than ionized donors [23].

Arsenic activation is typically achieved by high-low temperature annealing under mercury saturation. It is generally accepted that high-temperature annealing is performed at 400 °C or above, followed by low-temperature mercury compensation at 250 °C. The first high-temperature annealing step provides sufficient energy to break As-As bonds and enable arsenic site transfer (from the cation sublattice to the anion sublattice), while the second low-temperature annealing step serves to eliminate mercury vacancies generated during growth or annealing [9,21].

To investigate the effect of the arsenic doping concentration on the annealing results, samples 5#, 6#, and 7#, with different doping concentrations, were grouped, with sample 4# serving as the undoped intrinsic control. All of the samples were annealed under the previously optimized conditions of 420 °C/3 h + 250 °C/24 h. Additionally, to study the effects of different annealing atmospheres on crystalline quality and post-annealing electrical properties, samples 5#, 6#, and 7# were each divided into two subgroups: 5#-1, 6#-1, and 7#-1 and 5#-2, 6#-2, ad 7#-2, corresponding to two annealing atmospheres—one with pure Hg atmosphere and the other with Hg + CdTe atmosphere.

Table 3 presents the measurement results after annealing. It can be seen that arsenic doping is activated in all of the samples, and the conductivity type changes from n-type before annealing to p-type, achieving the goal of p-type doping activation. From the data in the table, when the arsenic doping concentration is below 5 × 10^17^ cm^−3^, the activation rate approaches 100%. As the As doping concentration increases, the activation rate shows a decreasing trend; when the impurity concentration reaches 8 × 10^18^ cm^−3^, the activation rate drops significantly to 26% (Hg atmosphere only). The analysis suggests that when the As concentration reaches a certain level, a large number of As_4_ cluster structures form in HgCdTe, which hinder As activation. Meanwhile, arsenic complexes or inactive arsenic clusters [24,25,26] in HgCdTe act as defects that differ from ionized donor scattering, scattering carriers and significantly reducing carrier mobility—the mobility of 7#-1 is approximately one-third that of 5#-1.

It can also be seen from Table 3 that the addition of CdTe along with Hg during annealing is highly beneficial for arsenic activation efficiency and carrier mobility. Sample 6#-1, annealed without CdTe, does not achieve full arsenic activation, whereas sample 6#-2, annealed with CdTe, achieves 100% activation. The effect is even more pronounced when comparing 7#-1 and 7#-2: the activation efficiency of 7#-2 is 2.5 times that of 7#-1, and the carrier mobility of 7#-2 is 5 times that of 7#-1. The analysis indicates that when only an Hg atmosphere is provided during annealing, HgCdTe decomposes at high temperatures, forming Te precipitates on the surface and resulting in poor crystalline quality. Dislocations within the material act as scattering centers, thereby reducing carrier mobility [27,28]. When both Hg and CdTe atmospheres are provided, CdTe decomposes slightly at high temperatures, establishing an equilibrium atmosphere of Cd vapor + Te_2_ vapor. This suppresses surface decomposition of HgCdTe, avoids surface Hg-rich or Te-rich conditions, inhibits excessive generation of mercury vacancies, promotes the formation of effective As_Te_ acceptors after As replaces Te sites, and reduces the formation of As_Hg_ by As occupying Hg sites. Studies suggest that, due to differences in lattice relaxation between As_Hg_ and V_Hg_, long-range coupling effects occur between them, and the resulting composite defects introduce multiple deep defect levels in the HgCdTe bandgap. Therefore, annealing activation under CdTe atmosphere protection not only improves arsenic doping activation efficiency [6,29] but also enhances carrier mobility.

## 4. Device Verification

Based on simulation results, a p-on-n heterojunction material with a p-type layer concentration of 5 × 10^17^ cm^−3^ was designed. This design concentration matches that of sample 6#, so As-doped HgCdTe heterojunction materials were epitaxially grown using the process conditions for sample 6#. Observation by scanning electron microscopy (SEM) revealed a smooth and flat material surface, as shown in Figure 6a. Statistical analysis of the surface macro-defect density was performed using an optical microscope at a magnification of 200×. We counted defects larger than 2 μm within each field of view, calculated the average defect count from multiple different fields of view, and then divided this average value by the area of a single field of view to obtain the macro-defect density. Statistical analysis showed a surface macro-defect density of 500 cm^−2^, indicating good material growth quality. High-Resolution X-ray Diffraction (HRXRD) was also used to measure the rocking curve of the material, yielding a full width at half maximum (FWHM) of 85 arcseconds (Figure 7), which further verifies the high quality of the epitaxial material. SIMS testing confirmed that the n-type layer of the heterojunction is indium-doped at 1 × 10^15^ cm^−3^, and the p-type layer is arsenic-doped at 5 × 10^17^ cm^−3^, as shown in Figure 6c. Since sample 6#-2 exhibited a good activation rate and carrier mobility after annealing, this annealing condition was selected as the activation annealing condition for the heterojunction material. After activation annealing, a 640 × 512 mesa array with a pixel pitch of 15 μm was fabricated using ICP etching and wet etching processes. Following passivation, annealing, contact hole etching, and electrode deposition, the HgCdTe heterojunction infrared detector array was finally obtained (Figure 6b). Figure 6d shows that the device exhibits excellent reverse breakdown characteristics (reverse breakdown voltage exceeding 800 mV), with a zero-bias resistance reaching 1 × 10^7^ Ω. After flip-chip bonding to the readout circuit and packaging, the detector maintains good performance at a high operating temperature of 110 K, with a noise equivalent temperature difference (NETD) of 21 mK and a blind pixel rate of only 0.2%. The response non-uniformity of the tested focal plane array (FPA) is 7.2%, which characterizes the homogeneity of the array. Figure 6 shows the spectral response of the detector, with the 50% cutoff of the maximum response at 5 μm (Figure 8). Conventional n-on-p detectors require operation at 77 K; by significantly increasing the operating temperature, this detector reduces fabrication costs and can also markedly improve reliability. Table 4 shows the benchmark of key performance metrics between this work and previously reported MBE-grown HgCdTe infrared FPAs on Si substrates.

## 5. Conclusions

As-doped Si-based HgCdTe materials were grown using an MBE system. The experiments show that the needle valve position of the As doping source has an approximately linear relationship with the arsenic beam flux, and the arsenic doping concentration in HgCdTe can be set by adjusting the needle valve position. The growth temperature of HgCdTe has a significant effect on the As doping concentration of epitaxial wafers: higher growth temperatures result in lower As doping concentrations. To maintain in-plane and longitudinal uniformity of arsenic doping in HgCdTe, it is necessary to ensure consistent in-plane temperature and consistent temperature throughout the growth process. During annealing activation, when the arsenic doping concentration is below 5 × 10^17^ cm^−3^, the activation rate approaches 100%; as the As doping concentration increases, both the activation rate and mobility decrease significantly. Annealing under Hg + CdTe atmosphere can markedly improve the arsenic doping activation rate and carrier mobility. Using MBE-grown HgCdTe heterojunction materials, 640 × 512 detector arrays with a pixel pitch of 15 μm were fabricated. The devices exhibit excellent reverse breakdown characteristics. The focal plane array after flip-chip bonding to the readout circuit achieves an NETD of 21 mK and a blind pixel rate of 0.2% at a high operating temperature of 110 K. The significant increase in operating temperature reduces detector fabrication costs. This work provides a reference for the engineering preparation of arsenic-doped HgCdTe heterojunction materials and offers guidance for the fabrication of low-cost, high-performance HgCdTe infrared detectors.

## Figures and Tables

**Figure 1:**
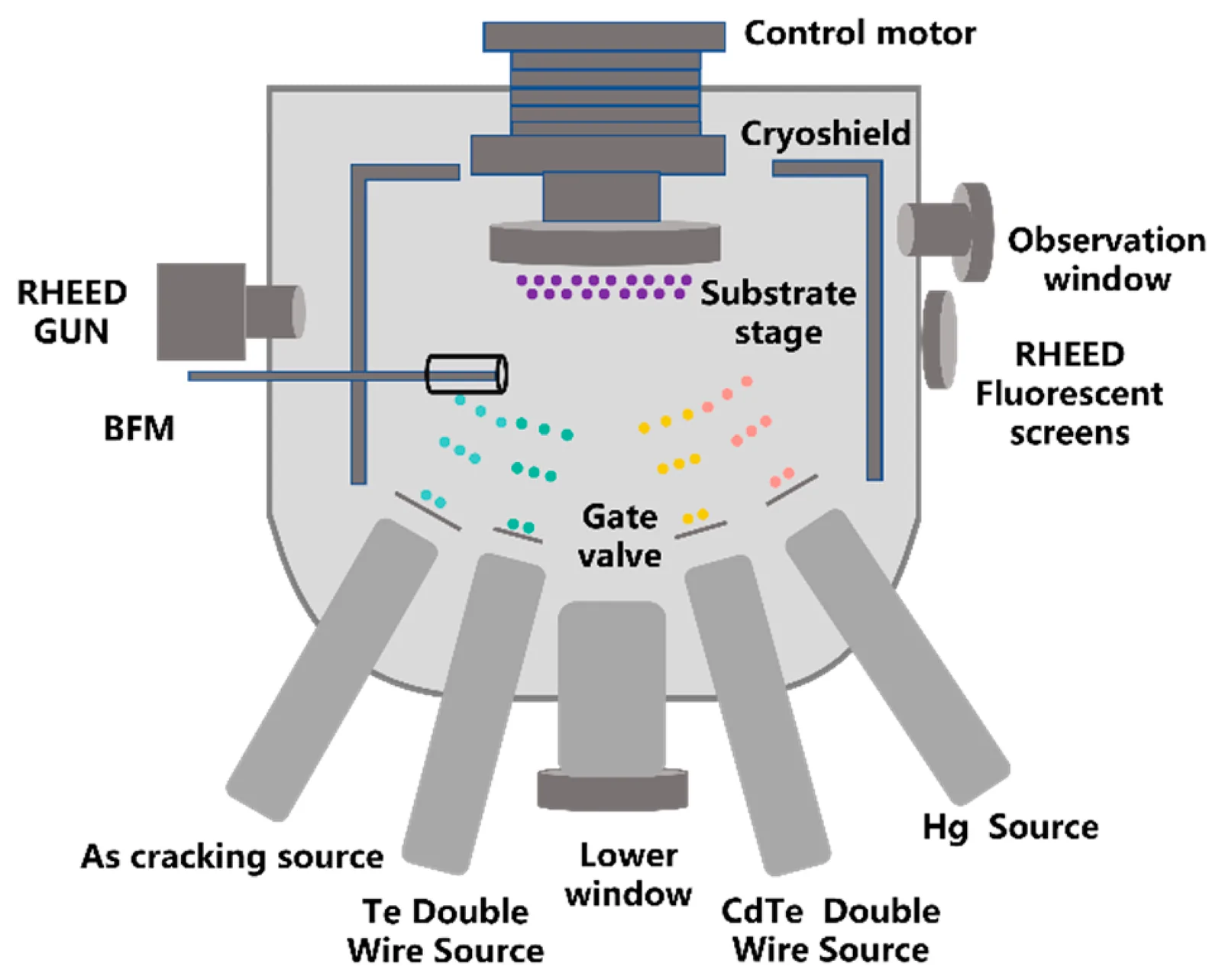
A schematic diagram of the molecular beam epitaxy system.

**Figure 2:**
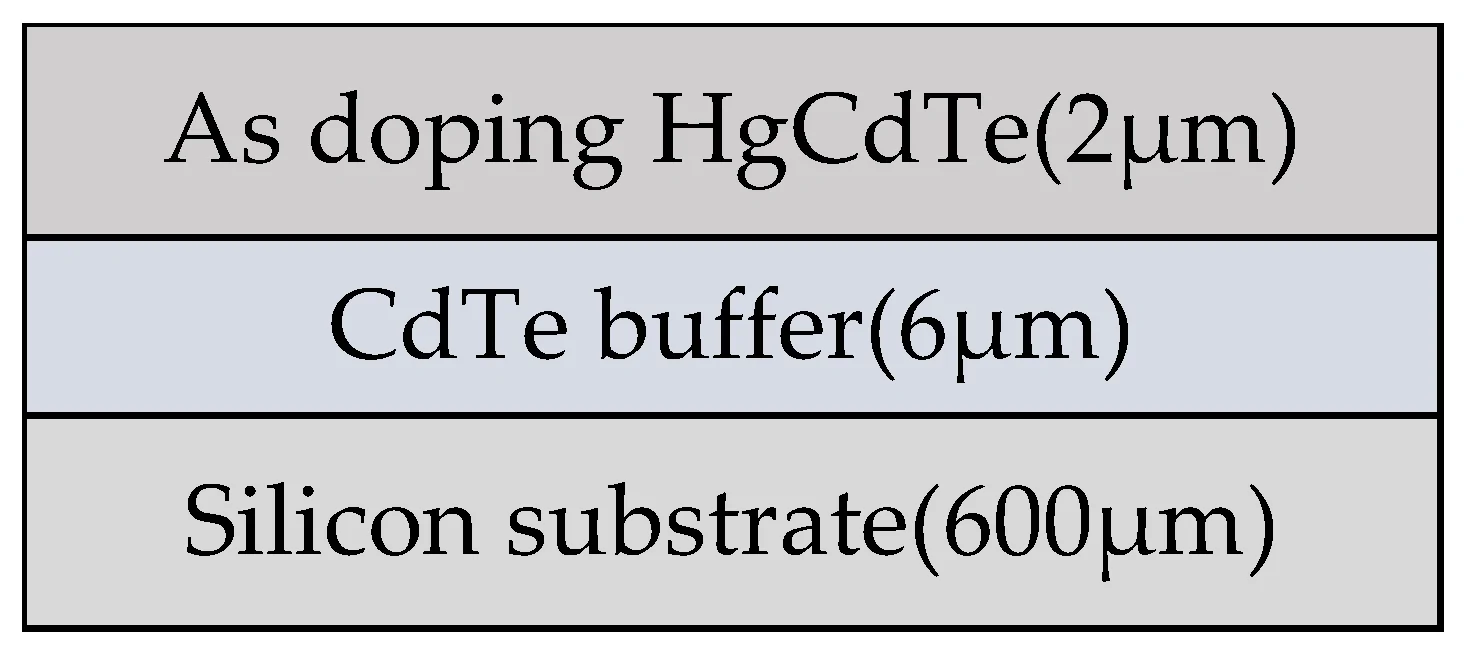
A schematic diagram of the arsenic-doped material structure.

**Figure 3:**
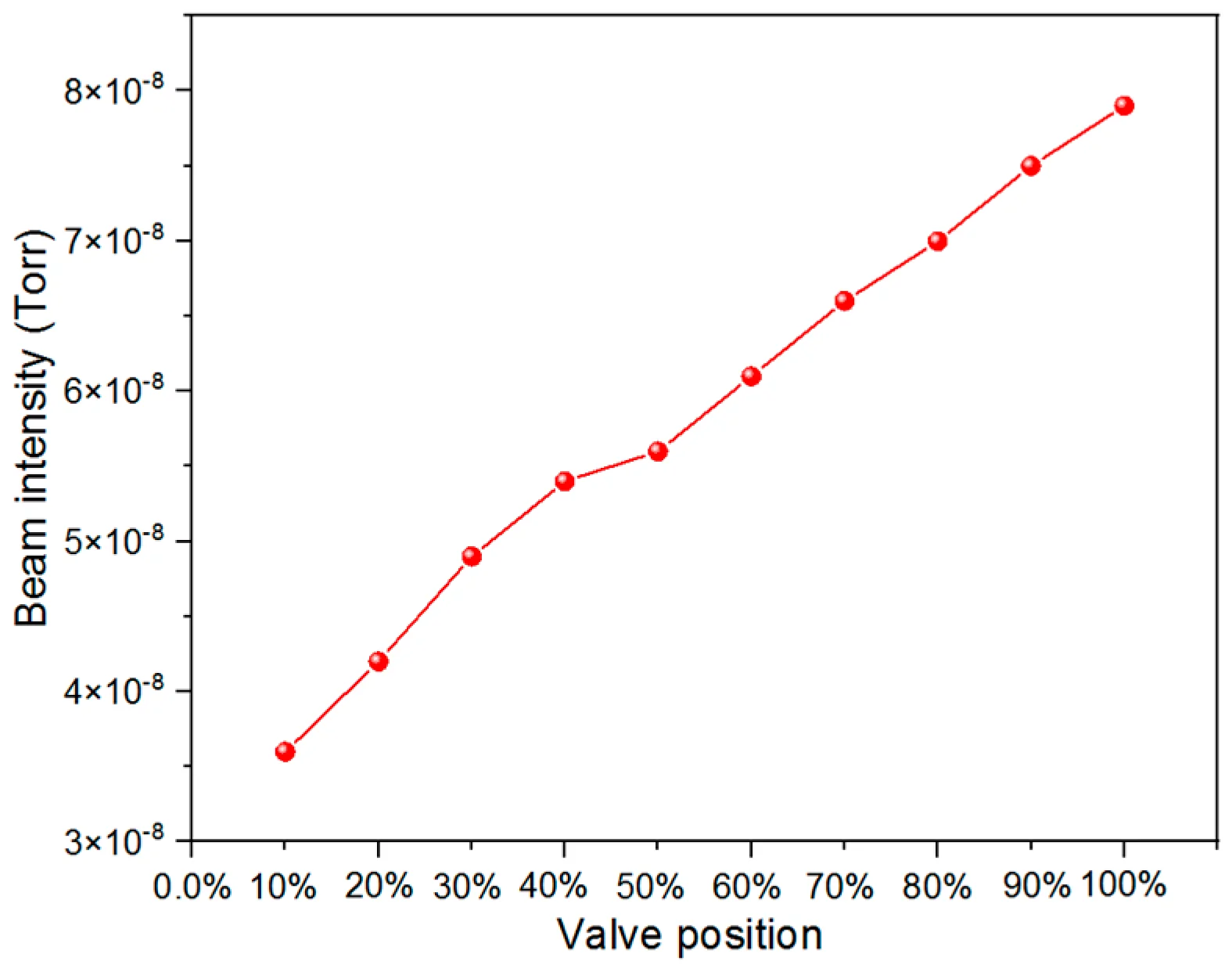
The relationship between the As source beam flux and the needle valve opening size. The relative measurement error of the beam flux is 5%.

**Figure 4:**
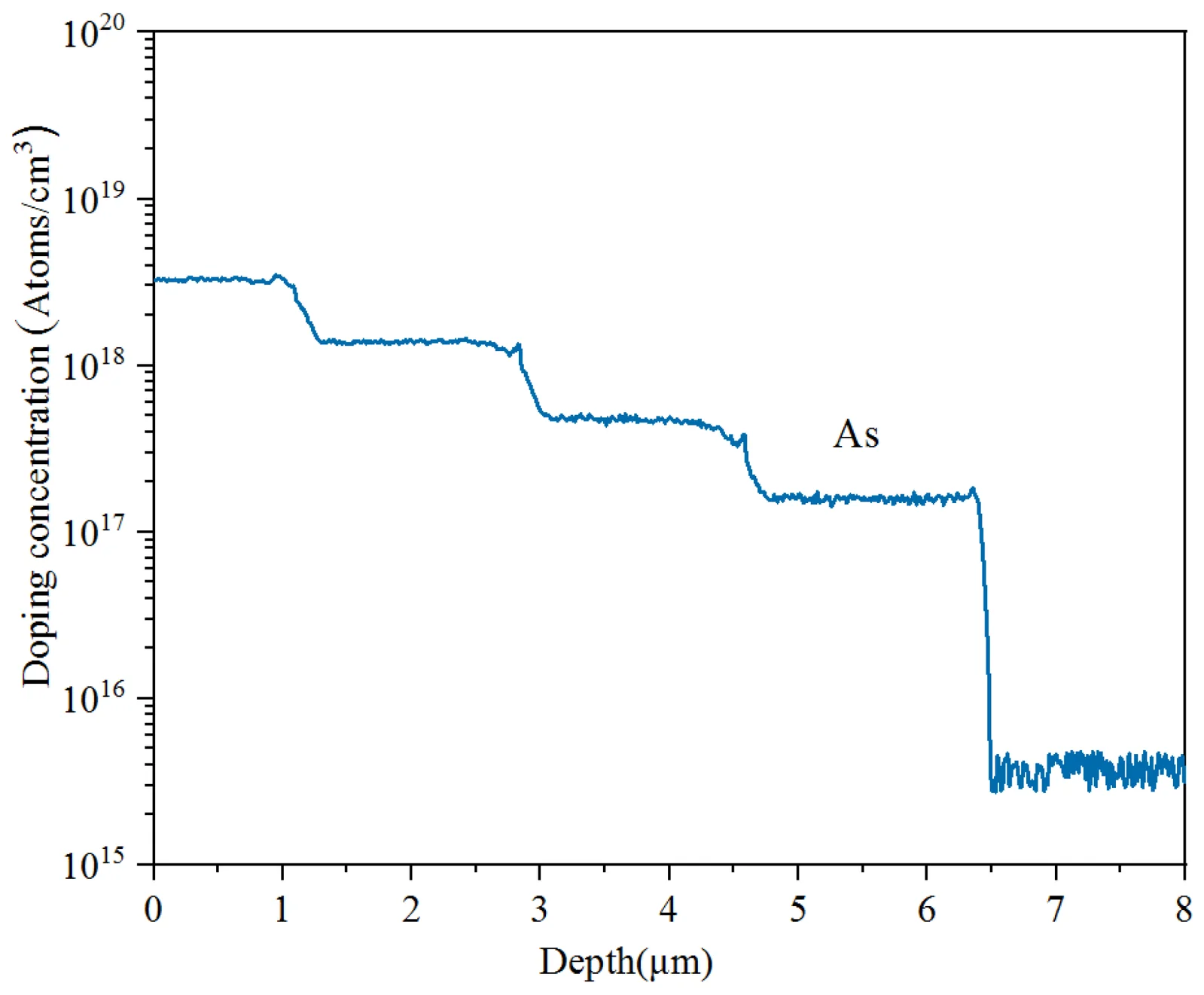
Relationship between As beam flux and As doping concentration.

**Figure 5:**
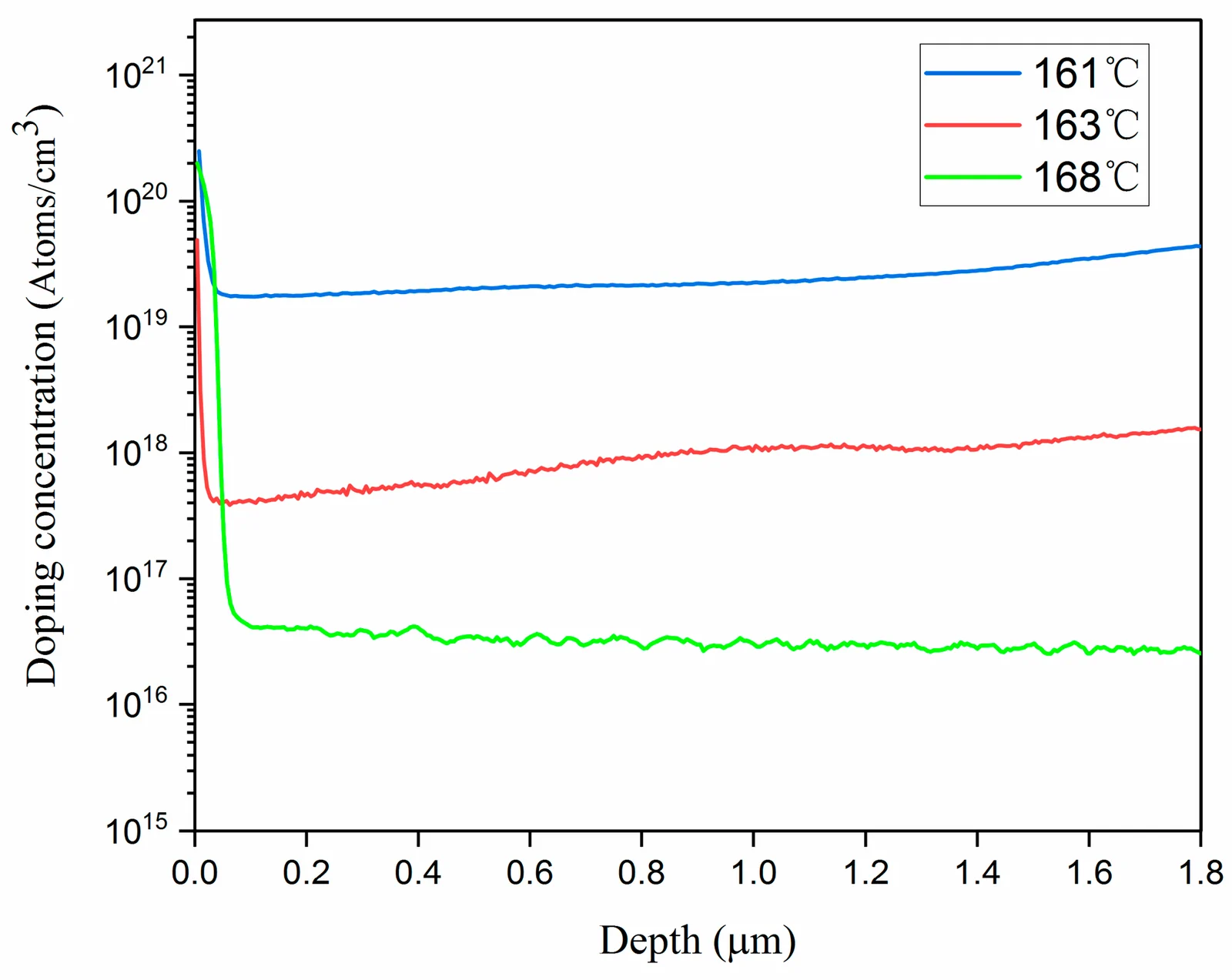
SIMS profiles of As-doped MCT materials grown at different temperatures.

**Figure 6:**
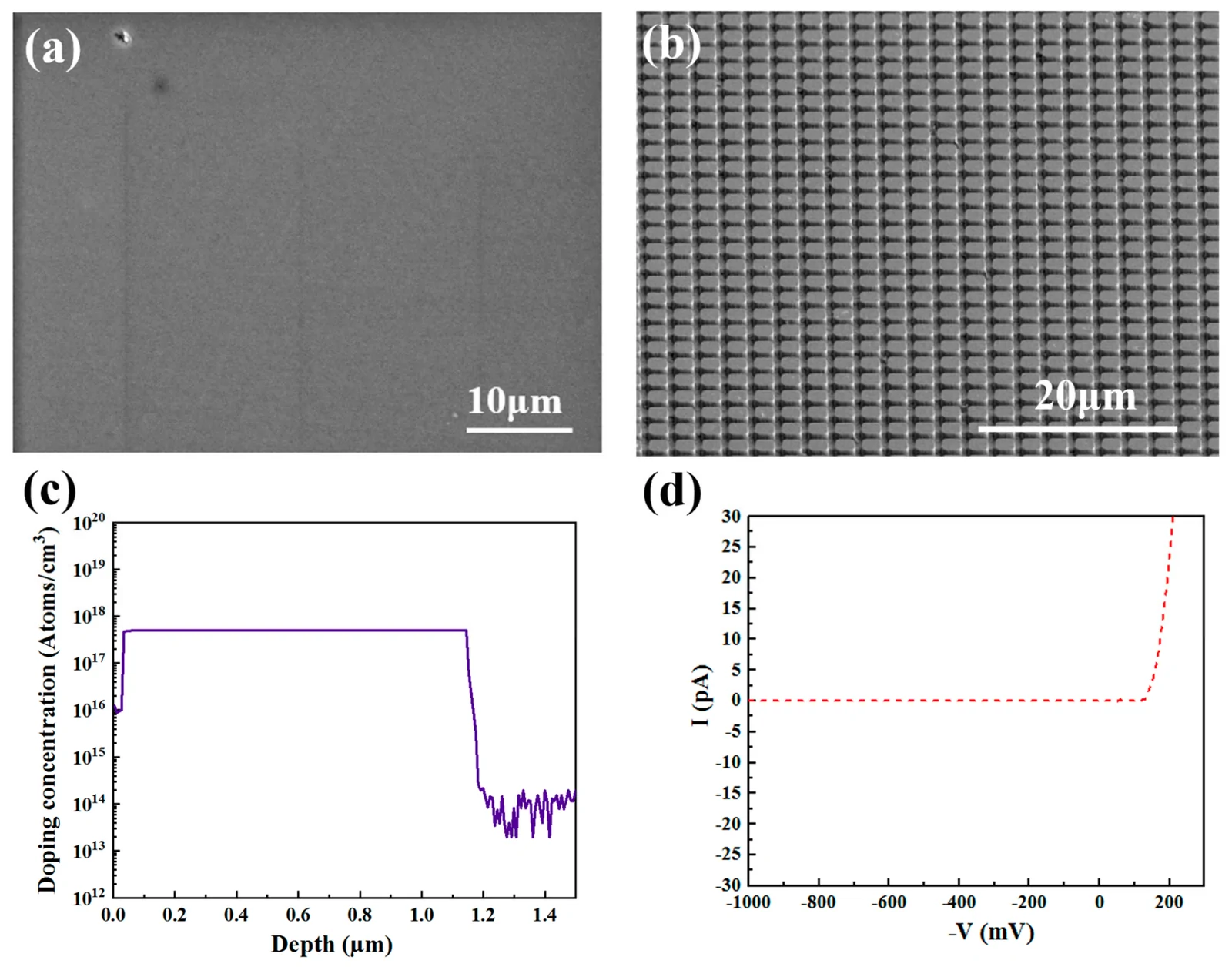
(**a**) Morphology of p-on-n heterojunction material; (**b**) HgCdTe heterojunction detector pixel array; (**c**) SIMS profile of As doping; and (**d**) I-V curve.

**Figure 7:**
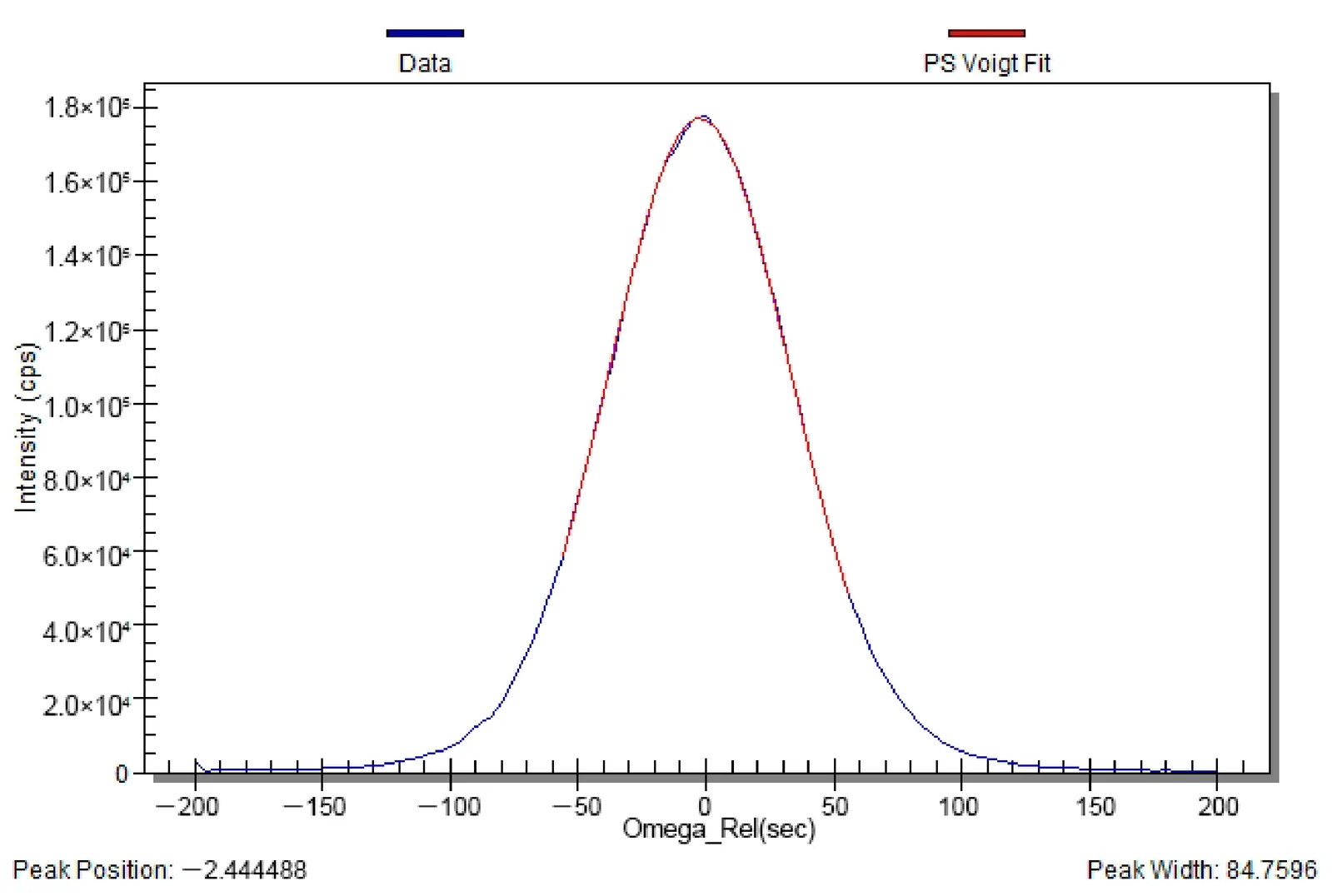
The HRXRD rocking curve of the HgCdTe heterojunction materials.

**Figure 8:**
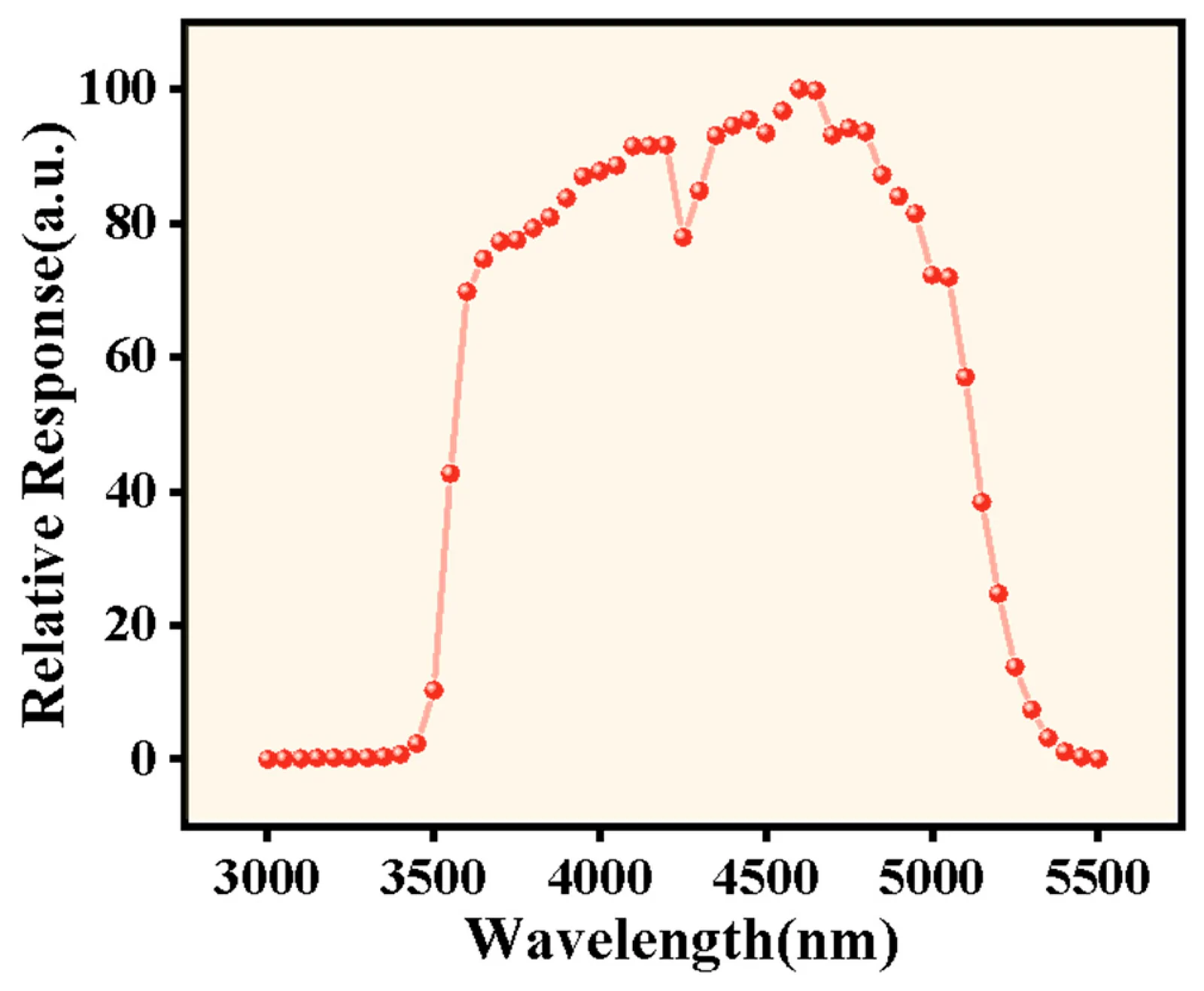
Spectral response.

**Table 1:** As-doped MCT grown at different temperatures.

Sample No.	Growth Temperature
1#	168 °C
2#	163 °C
3#	161 °C

**Table 2:** Hall test results of as-grown wafers with different As doping concentrations.

Sample No.	Conductivity Type	Carrier Concentration (cm^−3^)	Mobility (cm^2^·V^−1^·s^−1^)	Doping Concentration (cm^−3^)
4# (Control)	p	4.9 × 10^14^	356	--
5#	n	2.7 × 10^15^	8720.2	1 × 10^17^
6#	n	2.3 × 10^15^	4673.8	5 × 10^17^
7#	n	1.6 × 10^15^	4203.4	8 × 10^18^

**Table 3:** Hall test results after annealing with different As doping concentrations.

Sample No.	Atmosphere	Conductivity Type	Carrier Concentration (cm^−3^)	Mobility (cm^2^·V^−1^·s^−1^)	Activation Rate
5#-1	Hg	p	1.2 × 10^17^	38.1	100%
5#-2	Hg + CdTe	p	1.3 × 10^17^	225.6	100%
6#-1	Hg	p	4.9 × 10^17^	27.2	97.6%
6#-2	Hg + CdTe	p	5.1 × 10^17^	243.5	100%
7#-1	Hg	p	2.1 × 10^18^	13.6	26.4%
7#-2	Hg + CdTe	p	5.2 × 10^18^	68.1	65%

**Table 4:** Benchmark of key performance metrics between this work and previously reported MBE-grown HgCdTe infrared FPAs on Si substrates.

Parameter	This Work	Conventional	Ref. [30] (He et al., 2023)	Ref. [31] (Sellers et al., 2025)
Substrate	Si/CdTe	Si/CdTe	Si/CdTe	Si/CdTe
Architecture	p-on-n HgCdTe	n-on-p HgCdTe	p-on-n HgCdTe	nBn HgCdTe
Operating temperature	110 K	77 K	110 K	110 K
NETD	21 mK	23 mK	24 mK	28–33 mK
Blind pixel rate	0.2%	0.35%	0.32%	0.45%
Pixel-to-pixel non-uniformity	7.2%	6.5%	5.7%	6.8%

## Data Availability

All data in support of the findings of this paper are available within this article.
